# Profiling of intracellular metabolites produced from galactose and its potential for galactosemia research

**DOI:** 10.1186/s13023-018-0888-1

**Published:** 2018-08-24

**Authors:** Michel van Weeghel, Lindsey Welling, Eileen P. Treacy, Ronald J. A. Wanders, Sacha Ferdinandusse, Annet M. Bosch

**Affiliations:** 1 Laboratory Genetic Metabolic Diseases, Amsterdam UMC, University of Amsterdam, Amsterdam Gastroenterology & Metabolism, Amsterdam Cardiovascular Sciences, Meibergdreef 9, 1105 AZ Amsterdam, The Netherlands; 2 Department of Pediatrics, Emma Children’s Hospital, Amsterdam UMC, University of Amsterdam, Meibergdreef 9, 1105 AZ Amsterdam, The Netherlands; 3 National Centre for Inherited Metabolic Disorders, Temple St. Children’s University Hospital and Mater Misericordiae University Hospital, Dublin, Ireland; 4 Department of Pediatrics, room H7-270, Academic Medical Center, PO BOX 22660, 1100 DD Amsterdam, The Netherlands; 5 Department of Clinical Chemistry, Laboratory Genetic Metabolic Diseases, room F0-226, Academic Medical Center, PO BOX 22660, 1100 DD Amsterdam, The Netherlands

**Keywords:** Classical galactosemia, Galactose metabolites, Fibroblasts, UDP-galactose, Galactose-1-phosphate, Galactose oxidation

## Abstract

**Background:**

Clinical outcome of patients with a classical presentation of galactosemia (classical patients) varies substantially, even between patients with the same genotype. With current biomarkers, it is not possible to predict clinical outcome early in life. The aim of this study was to develop a method to provide more insight into galactose metabolism, which allows quantitative assessment of residual galactose metabolism in galactosemia patients. We therefore developed a method for galactose metabolite profiling (GMP) in fibroblasts using [U-^13^C]-labeled galactose.

**Methods:**

GMP analysis was performed in fibroblasts of three classical patients, three variant patients and three healthy controls. The following metabolites were analyzed: [U^13^C]-galactose, [U^13^C]-galactose-1-phosphate (Gal-1-P) and [^13^C_6_]- uridine diphosphate(UDP)-galactose. The ratio of [U^13^C]-Gal-1-P/ [^13^C_6_]-UDP-galactose was defined as the galactose index (GI).

**Results:**

All patient cell lines could be distinguished from the control cell lines and there was a clear difference between variant and classical patients. Variant patients had lower levels of [U^13^C]-galactose and [U^13^C]-Gal-1-P than classical patients (though substantially higher than healthy controls) and higher levels of [^13^C_6_]-UDP-galactose than classical patients (though substantially lower than healthy controls) resulting in a different GI in all groups.

**Conclusions:**

GMP in fibroblasts is a sensitive method to determine residual galactose metabolism capacity, which can discriminate between patients with a classical presentation of galactosemia, patients with a variant presentation and healthy controls. GMP may be a useful method for early prognostication after further validation in a larger cohort of patients representing the full phenotypic spectrum of galactosemia.

## Background

**Fig. 1:**
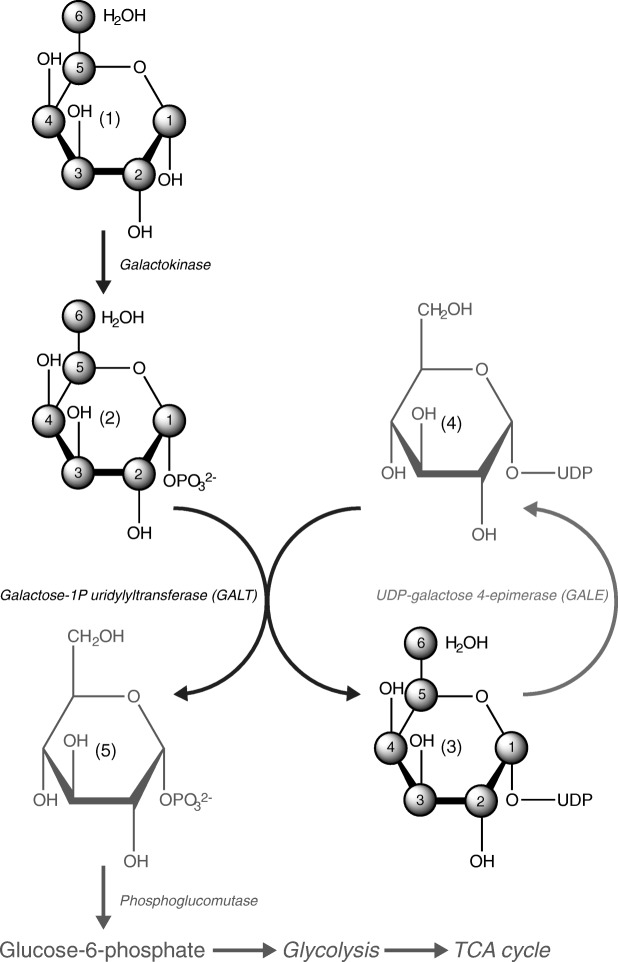
Schematic overview of galactose metabolism: Galactose [1] is converted by galactokinase (GALK1) to galactose-1-phosphate (Gal-1-P) [2] which is subsequently converted to uridine diphosphate (UDP)-galactose [3] by galactose-1-phosphate uridyltransferase (GALT). For this last conversion UDP-glucose [4] is used as a donor for the UDP and as a receptor for the phosphate to produce UDP-galactose [3] and glucose-1-phosphate [5]. Produced glucose-1-phosphate [5] can enter glycolysis via phosphoglucomutase to glucose-6-phosphate and subsequently enter the tricarboxylic acid (TCA) cycle. UDP-galactose [3] can be converted by UDP-galactose-4-epimerase (GALE) to UDP-glucose [4]. The numbered carbons are used in the galactose metabolites profiling (GMP) measurements

Deficiency of the enzyme galactose-1-phosphate uridyltransferase (GALT; EC 2.7.7.12) causes classical galactosemia (OMIM # 230400), an autosomal recessive inborn error of galactose metabolism. Despite early start of and good compliance with the galactose-restricted diet, which is the only available treatment, a substantial percentage of galactosemia patients suffers from burdensome complications, including decreased cognitive abilities, other neurological complications and primary ovarian insufficiency in females [1–5]. The mechanisms of disease are not fully understood yet. In the normal situation, GALT facilitates the conversion of galactose-1-phosphate (Gal-1-P) and uridine diphosphate (UDP)-glucose to glucose-1-phosphate and UDP-galactose (Fig. 1). Highly elevated levels of Gal-1-P in the fetus and newborn infant with galactosemia and persistently elevated Gal-1-P levels in patients even on dietary treatment (due to significant endogenous galactose production by the human body), are thought to play an important role in the pathophysiology. Elevated Gal-1-P levels have been demonstrated to competitively inhibit many metabolic pathways including glycosylation of proteins and lipids [6, 7]. As UDP-sugars are essential in the biosynthesis of glycoproteins and glycolipids [8, 9], a disturbed balance in UDP-sugars may also contribute to the glycosylation defects demonstrated in galactosemia [10]. The clinical outcome spectrum in galactosemia is highly variable, even in siblings with identical mutations and erythrocyte enzyme activities and ranges from fully normal to severely impaired development, which is poorly understood [11–14]. At this time, it is impossible to predict clinical outcome at the time of diagnosis based on the available biochemical, enzymatic and genetic data. Prognostic uncertainty is a major burden on parents and patients, especially since unnecessary treatment may occur and galactose over-restriction may even be harmful in some patients [15]. This is especially relevant since the extended newborn screening program (NBS) in the Netherlands has resulted in the identification of individuals with remarkable differences in biochemical and clinical phenotypes (henceforth called ‘variant patients’) compared to patients with a classical presentation of galactosemia (henceforth called ‘classical patients’) [16]. In this study none of the variant patients demonstrated any symptoms of galactosemia at the time of referral while they were on a galactose-containing diet until confirmation of the diagnosis, while 95% (19/20 patients) of classical patients demonstrated CG related illness at referral. At the time of the research these variant patients were all under the age of ten years, which explains why long-term clinical outcome was not reported. Biochemically they demonstrated markedly elevated Gal-1-P levels shortly after birth, comparable to classical patients. After introduction of a galactose-restricted diet, Gal-1-P levels showed an unusually rapid decrease (within six months) to the near undetectable range, while Gal-1-P levels remained significantly elevated in all treated classical patients. Some but not all variant patients had a higher residual erythrocyte GALT enzyme activity than patients diagnosed before NBS (up to 9% of healthy controls), and some had previously unreported genotypes. These children are thus referred to as having a variant (clinical and biochemical) presentation of galactosemia and most likely have a higher residual capacity to metabolize galactose. Furthermore, differences in galactose tolerance have been reported between classical patients [17]. In some of these patients improved glycosylation was observed with a somewhat increased galactose intake (using the IgG galactosylation marker). Thus, individualized prognostication is highly relevant and should ideally be followed by individualized treatment, which may improve outcome.

While at diagnosis the severity of GALT deficiency is determined by measurement of GALT activity in erythrocytes, the available enzyme assays have a number of limitations. First, the in vitro activity of GALT is not determined under physiological conditions, including saturated concentrations of the two substrates Gal-1-P and UDP-glucose. Second, they do not provide information on the effect of the enzyme deficiency on overall metabolism of galactose. The highly variable clinical outcome spectrum may also be a consequence of differences in the residual capacity to metabolize galactose in other tissues than the erythrocyte. In another inborn error of metabolism, very-long-chain acyl-CoA dehydrogenase (VLCAD) deficiency, fatty acid oxidation flux in fibroblasts was demonstrated to correlate much better with clinical severity than the enzyme activity in lymphocytes [18].

For all the above-mentioned reasons, we developed a method for galactose metabolites profiling (GMP) which provides information on galactose metabolism, by analyzing the intracellular levels of the metabolites generated from stable labeled [U^13^C]- galactose including Gal-1-P and UDP-galactose. We performed GMP in fibroblasts of classical patients and of patients with a variant presentation.

## Methods

### Patients

GMP was performed in fibroblasts of: 1) three classical patients (2 males, 1 female) who were diagnosed based on clinical presentation, 2) three variant patients (2 males, 1 female) who did not demonstrate any symptoms of illness at the time of diagnosis by NBS, and demonstrated a rapid decrease of Gal-1-P levels to undetectable (in 2) or just detectable (in 1) with a lactose free diet and 3) three healthy control subjects. Measurements were performed in fibroblasts which had been previously collected for clinical reasons. The Medical Ethics Review Committee of the AMC decided that the Medical Research Involving Human Subjects Act did not apply to this study and that their official approval was not required. All fibroblasts had been taken in early childhood.

### GALT enzyme activity and genetic analysis

Measurement of residual erythrocyte GALT enzyme activity and genetic analysis had been performed in all patients as part of the diagnostic work-up. Measurement of residual GALT enzyme activity in fibroblasts, essentially performed as described by Shin-Buehring [19], was performed as part of the diagnostic work-up in variant patients and for research purposes in the included classical patients.

### Cell culture procedure and stable isotope [^13^C]-labeled galactose metabolites profiling (GMP)

**Fig. 2:**
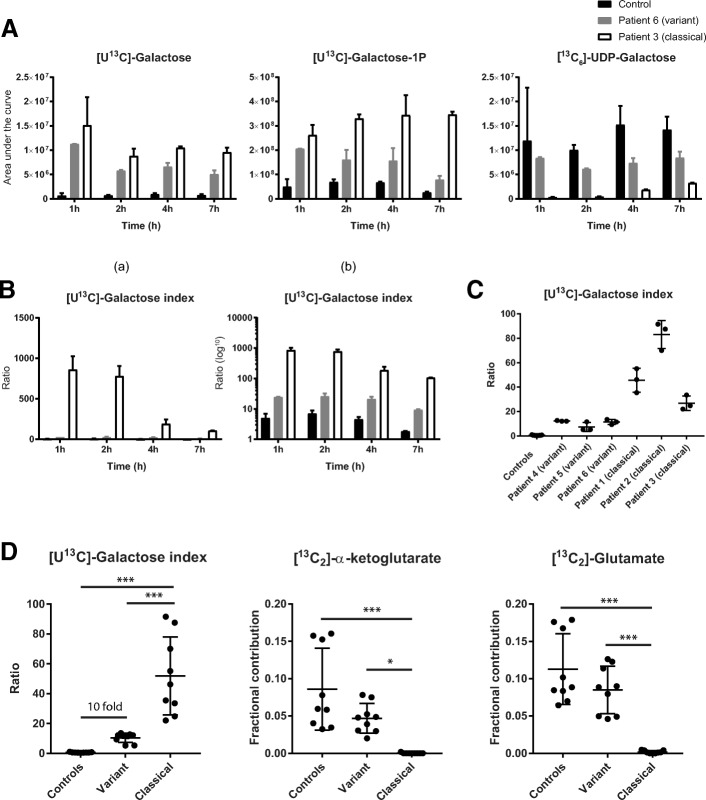
Stable [^13^C]-labeled galactose metabolism profiling (GMP) analysis: **a** Fibroblast metabolites of galactose metabolism incubated on different time points with [U^13^C]-galactose measured with mass spectrometry. **b** [U^13^C]-galactose index (GI) with Y-axis linear (a) and log10 (b). **c** The GI, measured after 7 h of incubation in cells of classical patients, variant patients and healthy controls. **d** Mean of the GI and downstream [^13^C]-labeled metabolites after 7 h incubation with [U^13^C]-galactose in cells of three healthy controls, three variant patients and three classical patients. Each experiment was performed independently three times. Significance is as follows: controls vs the mean of classical patients ***p < 0.001 and the mean of variant patients vs the mean of classical patients *p < 0.05, ***p < 0.001

For the stable [^13^C]-labeled GMP measurements, the metabolites of galactose including galactose, Gal-1-P and UDP-galactose were measured as output for GMP. Human fibroblasts were cultured at 37 °C under 5% CO_2_ in HAM F10 supplemented with 10% FCS and 100 μg/mL of penicillin/streptomycin. For the experiment, cells (50–100 μg of protein) were plated to 6 wells plates. After 24 h the medium was removed and cells were starved for 16 h using Dulbecco’s PBS without any extra additions. After 16 h of starvation, cells of healthy controls, classical patients and variant patients were incubated with 1 mM of [U^13^C]-galactose for 1 h, 2 h, 4 h or 7 h (Fig. 2a). Cells were washed three times with ice-cold saline solution (0.9%; *w*/*v*). Metabolism was quenched by adding 0,5 mL ice-cold methanol followed by 0,5 mL ice-cold water. For the extraction of metabolites, the 6 well plates were placed in a sonication bath and sonicated for 15 min. The homogenate was transferred to a 2 mL tube and after addition of 1 mL of chloroform, the homogenate was vortexed and centrifuged for 5 min at 18.620 rcf at 4 °C. The “polar” top layer was transferred to a new 1.5 mL tube and dried in a vacuum concentrator. Dried samples were dissolved in 100 μL methanol/water (6/4; *v*/v). The following metabolites were determined: [U^13^C]-galactose, [U^13^C]-Gal-1-P and [^13^C_6_]-UDP-galactose. The galactose index (GI) was defined as the ratio of [U^13^C]-Gal-1-P/[^13^C_6_]-UDP-galactose. For the analysis, we used a Thermo Scientific ultra-high-pressure liquid chromatography system (Waltman, MA, USA) coupled to a Thermo Q Exactive (Plus) Orbitrap mass spectrometer (Waltman, MA, USA). The autosampler was held at 10 °C during the runs and 5 μL of sample was injected on the analytical column. The chromatographic separation was established using a SeQuant ZIC-cHILIC column (PEEK 100 × 2.1 mm, 3.0 μm particle size, Merck, Darmstadt, Germany) and kept at 15 °C. The flow rate was 0.250 mL/min. The mobile phase was composed of (A) 9/1 acetonitrile/water with 5 mM ammonium acetate; pH 6.8 and (B) 1/9 acetonitrile/water with 5 mM ammonium acetate; pH 6.8, respectively. The LC gradient program was: beginning with 100% (A) hold 0–3 min; ramping 3–20 min to 36% (A); ramping from 20 to 24 min to 20% (A); hold from 24 to 27 min at 20% (A); ramping from 27 to 28 min to 100% (A); and re-equilibrate from 28 to 35 min with 100% (A). The MS data were acquired at full scan range, 140.000 resolution and in negative ionization mode. Interpretation of the data was performed in the Xcalibur software (Thermo scientific, Waltman, MA, USA).

### Statistical analysis

Statistical analysis was performed with Prism 7.02 (GraphPad, San Diego, CA, USA). Data are expressed as the means ± standard deviation (SD). Differences were evaluated with the one-way ANOVA multiple-comparisons test. When significant, the post hoc Bonferroni multiple-comparisons test was used to test differences between groups for significance. Statistical significance is indicated as detailed in the figure legends; *p*-values of ≤0.05 were considered significant. Isotope labeling correction was calculated using Mathworks Matlab (Natick, US).

## Results

### Patients

The three classical patients were homozygous for the common c.563A > G (p.Gln188Arg) mutation. Residual GALT enzyme activity in erythrocytes was below the limit of quantification of the enzyme assay in two patients and severely impaired in the third patient. The three variant patients had genotypes which were reported in our recent study [16] and had residual GALT enzyme activities in erythrocytes ranging from 4 to 9% of the mean of the reference values (Table 1).

**Table 1:** Genetic and biochemical characteristics of classical patients and variant patients

Patient	Sex	Genotype	Residual GALT enzyme activity in erythrocytes in μmol/(h.gram Hb) (reference value or range)	Residual GALT enzyme activity in fibroblasts in μmol/(h.mg protein) (reference range)
1 Classical	M	c.563A > G (p.Gln188Arg)/ c.563A > G (p.Gln188Arg)	< 0.5 (18–28)	< 0.01 (0.15–0.34)
2 Classical	M	c.563A > G (p.Gln188Arg)/ c.563A > G (p.Gln188Arg)	< 0.5 (18–28)	< 0.01 (0.15–0.34)
3 Classical	F	c.563A > G (p.Gln188Arg)/ c.563A > G (p.Gln188Arg)	0.25 (19.4) 0.36 (20.6)	< 0.01 (0.15–0.34)
4 Variant	F	c.563A > G (p.Gln188Arg)/ c.1-96 T > G	1.2 (21.8–44.9)	< 0.01 (0.15–0.34)
5 Variant	M	c.563A > G (p.Gln188Arg)/ c.656 T > A (p.Met219Lys)	2.4 (21.8–44.9)	0.04 (0.15–0.34)
6 Variant	M	c.382G > A (p.Val128IIe)/ c.382G > A (p.Val128IIe)	3.1 (21.8–44.9)	0.01 (0.15–0.34)

### Stable [^13^C]-labeled galactose metabolism profiling (GMP)

For setting up the GMP measurements we compared three groups (1 classical patient, 1 variant patient and 1 healthy control). [U^13^C]-galactose and [U^13^C]-Gal-1-P was increased in fibroblasts of the classical patient and variant patient, whereas [^13^C_6_]-UDP-galactose was decreased in these patients. This increase in [U^13^C]-Gal-1-P and decrease in [^13^C_6_]-UDP-galactose was time dependent (Fig. 2a). Furthermore, even the GI, defined as the ratio of [U^13^C]-Gal-1-P/ [^13^C_6_]-UDP-galactose, changed in time (Fig. 2b). The difference in GI between the different groups was most prominent for the shorter incubation times, however, the variations between the different measurements was the highest at 1 h. We observed that the most stable measurements were after 7 h of incubation and therefore we chose this time point as optimal incubation time (Fig. 2a and b).

The GI was then determined for all groups (3 individuals per group) and was increased in all patients compared to the healthy controls but was significantly higher in classical patients (*p* < 0.001) than in variant patients (10-fold change, one-way ANOVA multiple-comparisons test not significant) (Fig. 2c and d). These results show that the GI can be used as a measure of the severity of the GALT deficiency and residual galactose metabolism (Fig. 2c and d).

To establish whether galactose oxidation in fibroblasts from classical patients is truly fully impaired we also studied the incorporation of [^13^C]-labeled in other downstream metabolites including [^13^C]-labeled α -ketoglutarate and glutamate (Fig. 2d). In fibroblasts of classical patients there was essentially no formation of α-ketoglutarate and glutamate from [^13^C]-labeled galactose (*p* < 0.001), suggesting that galactose metabolism is blocked in fibroblasts of classical patients. In fibroblasts from variant patients however, α-ketoglutarate and glutamate were formed from [^13^C]-labeled galactose in comparable amounts to those found in control cells.

## Discussion

The aim of this study was to develop a method to provide more insight into galactose metabolism, which allows quantitative assessment of residual galactose metabolism in galactosemia patients. A method for GMP in fibroblasts using [U^13^C]-galactose as a substrate was developed, followed by metabolite analysis with tandem mass spectrometry. GMP analysis was performed in fibroblasts of patients with a classical presentation and a variant presentation of galactosemia.

Our results show that the developed GMP analysis is a sensitive method allowing discrimination of classical patients from variant patients, and the latter from healthy controls. These results indicate that variant patients have a higher residual capacity to metabolize galactose compared to classical patients and that the GI in fibroblasts (defined as the ratio of [U^13^C]-Gal-1-P/[^13^C_6_]-UDP-galactose) can be used as a measure for the severity of the GALT deficiency and residual galactose metabolism in galactosemia patients. The differences in GI between the three groups could already be observed after 1 h of incubation, though less variability between the different measurements and more significant differences between the groups were seen when measurements were performed at 7 h after incubation. For this reason, 7 h was chosen as optimal incubation time. Before implementation in the diagnostic workup, the developed GMP analysis in fibroblasts needs further validation in a larger group of galactosemia patients representing the whole clinical outcome spectrum. It is essential to determine if patients with a classical presentation, but with different long-term outcomes, have different profiles of galactose metabolites and if GMP can thus be used as predictor of outcome.

With the current method for GMP, we could not differentiate [^13^C_6_]-UDP-galactose from [^13^C_6_]-UDP-glucose and [U^13^C]-Gal-1-P from [U^13^C]-glucose-1-phosphate. For the current study this is only a small limitation, as the labeled substrate first has to pass the GALT enzyme before it can be detected either as [^13^C_6_]-UDP-galactose, [^13^C_6_]-UDP-glucose or [U^13^C]-glucose-1-phosphate. If the GALT deficiency is more severe, less of the substrate will pass this step and less [^13^C_6_]-UDP-galactose and [^13^C_6_]-UDP-glucose will be detected, which is clear from the differences between classical patients and variant patients. A next step in future research will be to discriminate [^13^C_6_]-UDP-galactose from [^13^C_6_]-UDP-glucose and discriminate [U^13^C]-Gal-1-P from [U^13^C]-glucose-1-phosphate in this analysis. This may be important because in classical patients, differences in residual capacities to metabolize galactose affecting clinical outcome may be smaller. Furthermore, if [^13^C_6_]-UDP-galactose can be separated from [^13^C_6_]-UDP-glucose, UDP-glucose 4-epimerase (GALE) deficiency can also be studied.

## Conclusions

Galactose metabolites profiling (GMP) in fibroblasts is a sensitive method to determine residual galactose metabolism capacity which can discriminate between patients with a classical presentation of galactosemia, patients with a variant presentation and healthy controls. The results in this research indicate that variant patients have a higher residual capacity to metabolize galactose compared to classical patients. The developed GMP analysis may be a good method for early prognostication of individuals with GALT deficiency, though this method should be further validated in a larger group of individuals with several degrees of GALT deficiency representing the full outcome spectrum.
